# TLR activation enhances C5a-induced pro-inflammatory responses by negatively modulating the second C5a receptor, C5L2

**DOI:** 10.1002/eji.201041350

**Published:** 2011-06-01

**Authors:** Anne-Catherine Raby, Benjamin Holst, James Davies, Chantal Colmont, Yves Laumonnier, Barbara Coles, Sanjoy Shah, Judith Hall, Nicholas Topley, Jörg Köhl, B Paul Morgan, Mario O Labéta

**Affiliations:** 1 Department of Infection, Immunity and Biochemistry; School of Medicine, Cardiff UniversityCardiff, UK; 2 Department of Anaesthetics and Intensive Care Medicine, School of Medicine, Cardiff UniversityCardiff, UK; 3 Institute for Systemic Inflammation Research, University of LübeckLübeck, Germany; 4 Division of Molecular Immunology, Cincinnati Children's Hospital Medical Center and University of Cincinnati College of MedicineOH, USA

**Keywords:** Complement, Inflammation, Innate immunity, TLR

## Abstract

TLR and complement activation ensures efficient clearance of infection. Previous studies documented synergism between TLRs and the receptor for the pro-inflammatory complement peptide C5a (C5aR/CD88), and regulation of TLR-induced pro-inflammatory responses by C5aR, suggesting crosstalk between TLRs and C5aR. However, it is unclear whether and how TLRs modulate C5a-induced pro-inflammatory responses. We demonstrate a marked positive modulatory effect of TLR activation on cell sensitivity to C5a in vitro and ex vivo and identify an underlying mechanistic target. Pre-exposure of PBMCs and whole blood to diverse TLR ligands or bacteria enhanced C5a-induced pro-inflammatory responses. This effect was not observed in TLR4 signalling-deficient mice. TLR-induced hypersensitivity to C5a did not result from C5aR upregulation or modulation of C5a-induced Ca^2+^ mobilization. Rather, TLRs targeted another C5a receptor, C5L2 (acting as a negative modulator of C5aR), by reducing C5L2 activity. TLR-induced hypersensitivity to C5a was mimicked by blocking C5L2 and was not observed in C5L2KO mice. Furthermore, TLR activation inhibited C5L2 expression upon C5a stimulation. These findings identify a novel pathway of crosstalk within the innate immune system that amplifies innate host defense at the TLR-complement interface. Unravelling the mutually regulated activities of TLRs and complement may reveal new therapeutic avenues to control inflammation.

## Introduction

The innate immune system plays a crucial role in the inflammatory response to infection through the activity of receptors capable of recognizing defined molecular patterns present in a variety of microorganisms. In particular, the concerted activity of two components of the innate immune system, TLRs and complement, results in rapid inflammatory responses and also orchestrates adaptive immune responses that lead to clearance of infection 1–4.

TLRs are critical to the triggering of the inflammatory response. They recognize and respond to an array of microorganisms and their components, many of which can also activate complement, by mediating a prompt and efficient pro-inflammatory response 1, 5. This includes the production of a variety of inflammatory mediators, e.g. IL-6, TNF-α, IL-8 (CXCL8), MCP-1 (CCL2), resulting in an immediate response to the microbial challenge. However, the excessive release of pro-inflammatory molecules as a consequence of TLR hyperactivation can lead to serious pathological conditions such as acute inflammation, tissue/organ damage, septic shock, chronic inflammation and autoimmunity 6, 7.

Microorganisms and their components also activate the complement system, which plays a significant role in acute inflammation and the destruction of invading microorganisms. Complement activation leads to the generation of biologically active complement peptide fragments such as C5a and C3a that elicit a number of pro-inflammatory effects 2, 8. The complement anaphylatoxin C5a in particular is one of the most potent pro-inflammatory peptides. It acts as a granulocyte, monocyte and macrophage chemoattractant. It is a vasodilator, induces the oxidative burst in neutrophils and enhances phagocytosis, granule enzyme release and adhesion molecule expression. It activates the coagulation cascade, induces the synthesis and release of arachidonic acid metabolites as well as pro-inflammatory cytokines and chemokines. Excessive generation of C5a, however, contributes to serious inflammatory conditions such as sepsis 9, 10. C5a exerts most of its effects through the C5a receptor (C5aR), a seven-transmembrane G protein-coupled receptor. Recently, the involvement of a second seven-transmembrane, but G protein-uncoupled, receptor for C5a, C5L2, in the biological activities of C5a and as a negative modulator of C5aR activity has been reported 11–13.

Given the serious acute and chronic inflammatory conditions resulting from over-activation or dysregulation of TLR-mediated responses and/or excessive C5a-induced pro-inflammatory responses, TLR and C5a receptor signalling are attractive therapeutic targets for the treatment and/or prevention of inflammatory conditions 7, 9, 14–16. Therefore, understanding the mechanisms modulating TLR activity and the C5a-C5a receptor interaction is of major interest.

Notably, it has previously been shown that cell exposure to a combination of LPS and C5a resulted in enhanced production of a number of cytokines and chemokines, indicating synergism between TLRs and C5aR 17–19. Furthermore, C5a-triggered signalling through C5aR substantially enhanced or modulated microbial-induced pro-inflammatory cytokine production mediated by TLRs 20, 21. A negative effect of C5aR engagement on the TLR4-mediated production of the IL-12 family of immunomodulatory cytokines has also been reported 22, and recently it has been demonstrated that *Porphyromonas gingivalis*-generated C5a enhancement of TLR2-mediated cyclic AMP production results in macrophage immunosuppression 23. Thus, through the C5a–C5aR interaction, complement appears to influence the extent of the pro-inflammatory and immunomodulatory responses triggered via TLRs. Together, these findings provided evidence for crosstalk between the complement system and TLRs, strengthening the innate host defense during infection 3, 4. However, most studies have focused on the immunoregulatory effect of C5a on TLR-driven inflammation, neglecting potential effects of TLR activation on complement-mediated inflammation. In the present study, we have therefore evaluated the impact of TLR activation on C5a-driven cytokine and chemokine production in vitro and ex vivo. Our data demonstrate a marked positive modulatory effect of TLR activation on cell sensitivity to C5a, supporting the concept of a genuine crosstalk between TLRs and C5aR, and indicate that TLRs may exert their modulatory effect by reducing the negative regulatory capacity of C5L2 on C5aR.

## Results

### PBMCs pre-exposed to TLR ligands are hypersensitive to C5a stimulation

**Figure 1:**
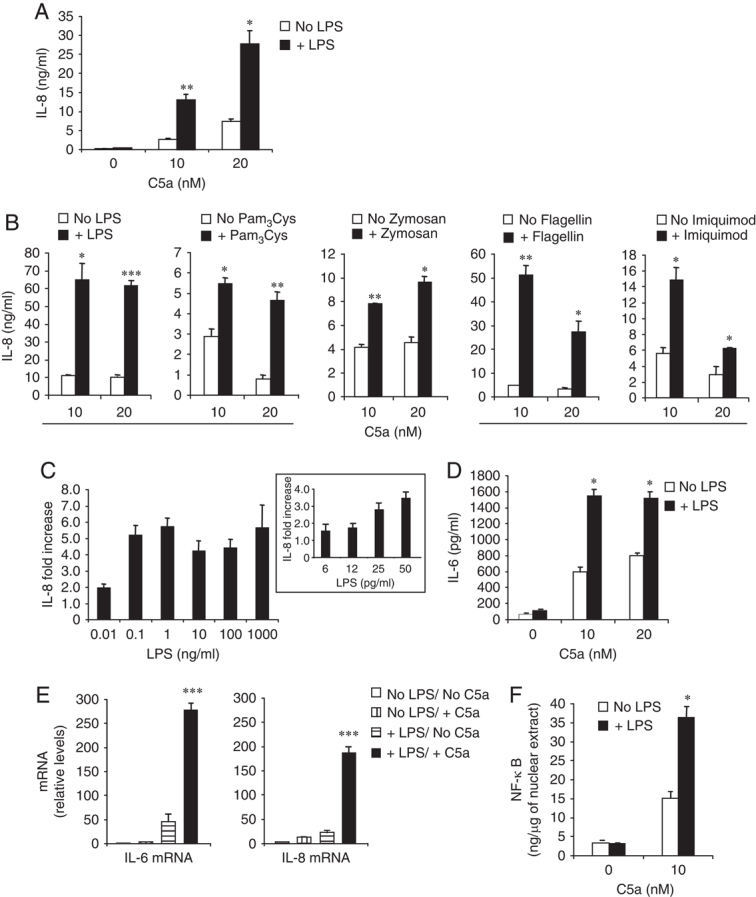
Sensitivity to C5a of PBMCs pre-exposed to TLR ligands. (A–C) Levels of IL-8 in culture supernatants of PBMCs (1.5×10^5^/well) (A) stimulated for 14 h with the indicated concentrations of C5a and (B) after washing and re-culture following pre-exposure (14 h) to LPS (100 pg/mL or as indicated), Pam_3_Cys (100 ng/mL), Zymosan (1 μg/mL), Flagellin (5 μg/mL), Imiquimod (3 μg/mL) or mock-pre-exposure (no TLR ligand). IL-8 concentrations were estimated by subtracting the background levels of IL-8 present in cultures not activated with C5a and pre-exposed or not to TLR ligands from the corresponding C5a-activated samples (IL-8 background levels (ng/mL): No ligand/No C5a, 1.6±0.7; +LPS, 2.3±1.2; +Pam_3_Cys, 1.5±0.6; +Zymosan, 1.3±0.9; +Flagellin, 6.9±2.5; +Imiquimod, 5.3±1.1; *n*≥4). (C) IL-8 fold increases were determined by comparing IL-8 levels – after background subtraction – between C5a-stimulated (10 nM) cell samples pre-exposed and not pre-exposed to LPS. (D) Levels of IL-6 in culture supernatants of PBMCs stimulated for 14 h with the indicated concentrations of C5a, after washing and re-culture following pre-exposure to LPS. (E and F) Determination of (E) IL-6 and IL-8 mRNA levels in RNA samples and (F) NF-κB concentrations in the nuclear extracts of PBMCs pre-exposed or not to LPS and subsequently stimulated with C5a as described for A–D. (E) mRNA levels are relative to control (No LPS/No C5a). Results are from one experiment (+SD) representative of at least four for each ligand (A, B) or three (C–F). ^*^p<0.05, ^**^p<0.01, ^***^p<0.005 (TLR-pre-exposed versus TLR not pre-exposed, paired Student's *t*-test).

To evaluate a potential modulatory effect of TLRs on C5a-mediated pro-inflammatory responses, PBMCs were first stimulated with TLR ligands. Following extensive washing, the cells were stimulated with C5a before assessment of IL-8 levels in the cell culture supernatants. Figure 1A shows that the levels of IL-8 released by C5a-stimulated PBMCs pre-exposed to the TLR4 ligand, LPS, were substantially higher than those released by cells not pre-exposed to LPS. Similarly, a two- to ten-fold increase in the C5a-induced release of IL-8 was observed when PBMCs were pre-exposed to ligands for TLR2/TLR1, TLR2/TLR6 (the synthetic bacterial lipopeptide, Pam_3_-Cys-Ser-Lys_4_ and yeast zymosan respectively), TLR5 (bacterial flagellin) and TLR7/TLR8 (the antiviral compound, imiquimod), the extent of the increase depending on the TLR ligand and the C5a concentration tested (Fig. 1B). TLR-induced hypersensitivity to C5a was observed following pre-exposure to a wide range of TLR ligand concentrations, as is shown in Fig. 1C in the case of LPS. Here, even pre-exposure to 10 pg/mL LPS, a concentration well below those found in sepsis patients (∼100 pg/mL to ∼700 pg/mL, 24), resulted in hypersensitivity to C5a. Together, these findings indicated that TLR activation imparts hypersensitivity to blood mononuclear cells to C5a.

TLR-induced hypersensitivity to C5a was not restricted to the release of IL-8, as the release of the pro-inflammatory cytokine, IL-6, was similarly enhanced (Fig. 1D). Furthermore, PBMC pre-exposure to TLR ligands not only affected the C5a-induced release but also the transcription of pro-inflammatory mediators, since mRNA levels for both IL-6 and IL-8 markedly increased (Fig. 1E). Consistent with this finding, the activation levels of the transcription factor NF-κB – a key regulator of immunoregulatory gene transcription – in nuclear extracts of C5a-stimulated PBMCs that were pre-exposed to LPS were substantially higher than those in cells not pre-exposed (Fig. 1F). This finding also indicated that TLR modulation of cell sensitivity to C5a has a wide spectrum of activities and, thus, a wide range of pro-inflammatory and immunomodulatory mediators might be affected.

### Whole blood pre-exposure to LPS or *Escherichia coli* increases blood cell sensitivity to C5a

**Figure 2:**
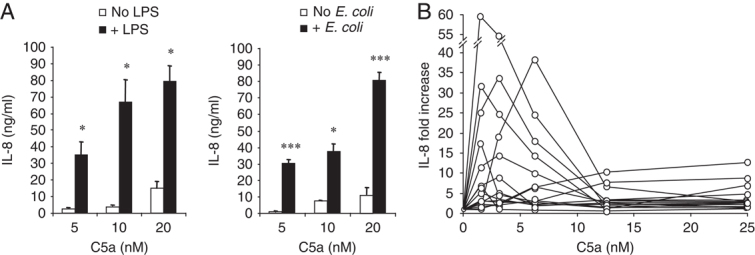
Sensitivity to C5a of human blood cells pre-exposed to LPS or *E. coli*. (A) C5a-induced levels of IL-8 and (B) fold increase in IL-8 concentration in blood cell culture supernatants following whole blood (100 μL/well) pre-exposure or not to LPS (500 pg/mL, A, left and B) or *E. coli* (1×10^8^ CFU/mL). Pre-exposure to TLR ligands followed by C5a stimulation, and estimation of C5a-induced IL-8 concentrations and fold increases were as described for Fig. 1. (A) Results are from one experiment (+SD) representative of three. ^*^p<0.05, ^***^p<0.005 (LPS- or *E. coli*-treated versus mock-treated, paired Student's *t*-test). (B) Response profile of 15 healthy blood donors.

To better evaluate the in vivo relevance of the positive modulatory effect of TLR activation on cell sensitivity to C5a, we used a minimally perturbed experimental model in which human whole blood was pre-exposed to LPS or whole *E. coli*. Following washing, the blood cells were resuspended in autologous plasma and stimulated with varying concentrations of C5a. Pre-exposure of whole blood to LPS or whole bacteria resulted in a substantially higher sensitivity of blood cells to C5a stimulation (Fig. 2A), suggesting that TLR modulation of peripheral blood immunocompetent cell sensitivity to C5a might occur in vivo. Analysis of the modulatory effect over a wide range of C5a concentrations in 15 blood donors showed that the extent of the increase in cell sensitivity to C5a depended on both the donor and the concentration of C5a tested (Fig. 2B), with 13 out of the 15 donors showing a decline in their response to TLR activation (lower hypersensitivity to C5a) at relatively high C5a concentrations.

### LPS-induced enhanced blood cell sensitivity to C5a is not observed in TLR4 signalling-deficient mice

**Figure 3:**
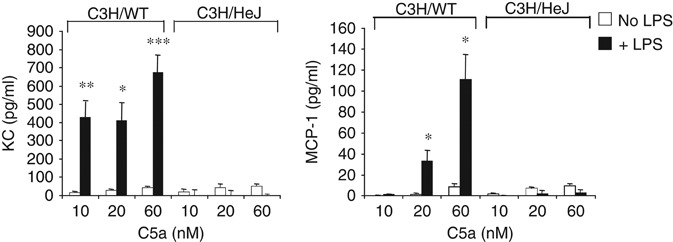
Ex vivo blood cell sensitivity to C5a of TLR4 signalling-deficient and WT mice. KC and MCP-1 levels in blood cell culture supernatants (100 μL whole blood/condition) of C3H/HeJ and C3H/HeN (WT) mice stimulated (14 h) ex vivo with C5a following a challenge (1 h) i.p. with LPS (50 μg/mouse) or PBS (no LPS). The C5a-induced chemokine concentrations were estimated by background subtraction as described for Fig. 1. Values are expressed as the mean+SEM (*n*=5/condition).^*^p<0.05, ^**^p<0.01, ^***^p<0.005 (LPS-treated versus no LPS, paired Student's *t*-test).

To demonstrate that microbial-induced cell hypersensitivity to C5a strictly depended on TLR activation, we compared blood cell sensitivity to C5a ex vivo between mice deficient in TLR4 signalling (C3H/HeJ) and WT (C3H/HeN) mice that had been challenged with LPS (Fig. 3). The C5a-induced release of the prototypical polymorphonuclear and mononuclear cell chemoattractants keratinocyte-derived chemokine (KC, a murine functional counterpart of human IL-8) and MCP-1 (CCL2), respectively, was extremely low in both TLR4-deficient and WT mice that had not been previously challenged with LPS. However, pre-exposure to LPS resulted in a markedly higher blood cell sensitivity to C5a in WT, but not in TLR4-deficient mice (Fig. 3), thus confirming the crucial role that TLR activation plays in this phenomenon and supporting the in vivo relevance of the modulatory effect of TLRs.

### Pre-exposure to LPS downregulates C5aR expression

**Figure 4:**
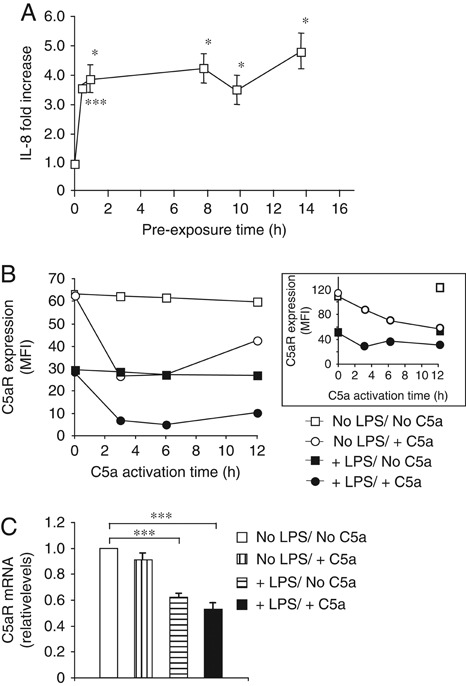
Effect of a pre-exposure to LPS on cell sensitivity to C5a and C5aR expression. (A) Fold increase in the levels of IL-8 – determined as described for Fig. 1– in culture supernatants of PBMCs (1.5×10^5^/well) pre-exposed or not to LPS (100 pg/mL) for the indicated times (starting from 30 min), and subsequently activated (14 h) with C5a (10 nM). (B) C5aR cell-surface expression levels on gated monocytes or neutrophils (inset) at different times following 1×10^6^ PBMC (monocytes) or 100 μL whole blood (neutrophils) pre-exposure (30 min) to 100 pg/mL (PBMCs) or 500 pg/mL (whole blood) LPS or a mock-pre-exposure (no LPS), and subsequent activation or not with C5a (10 nM) for the indicated times. (C) C5aR mRNA levels determined by RT-qPCR in RNA samples extracted from PBMC aliquots taken from the experiment described in (B) at the end of the culture (12 h). Results are from one experiment (A and C, ±SD) representative of three. ^*^p<0.05, ^***^p<0.005 (LPS-pre-exposed versus not pre-exposed, paired Student's *t*-test).

The positive modulation exerted by TLRs posed the question of the underlying mechanism. The TLR modulatory effect appears to be relatively rapid, as a 30-min pre-exposure to LPS was sufficient to achieve maximal hypersensitivity to C5a (Fig. 4A). We then tested whether TLRs modulate cell sensitivity to C5a by upregulating C5aR expression. Given that monocytes are the main TLR-expressing cell type in leukocytes 25, the main target for C5a in PBMCs, and that they orchestrate many of the TLR-induced responses of peripheral blood leukocytes, including those of neutrophils 25, we focused on monocyte C5aR and monitored its expression in PBMCs after a 30-min pre-exposure to LPS. Subsequently, we continued monitoring C5aR expression following stimulation with C5a. Monocyte C5aR cell-surface expression was markedly lower in PBMCs pre-exposed to LPS (Fig. 4B, left, +LPS/No C5a versus No LPS/No C5a). After 3 h of C5a stimulation, both LPS-treated and untreated monocytes showed lower cell-surface levels of C5aR than those at time 0. By 12 h post-C5a stimulation, C5aR levels had partially recovered. This modulation pattern of C5aR expression following C5a stimulation most likely reflects C5a-induced receptor internalization and its recycling back to the cell surface, as previously described 26–28. In parallel experiments, we confirmed that PBMCs from the same donors showed hypersensitivity to C5a (IL-8 (ng/mL): No LPS/No C5a, 0.9±0.3; No LPS/+C5a, 3.2±1.1; +LPS/No C5a, 1.9±0.4; +LPS/+C5a, 16.9±3.5; *n*=3). The LPS-induced downmodulation of C5aR cell-surface expression correlated with the determinations of C5aR mRNA expression in PBMC aliquots collected from the experiment described above at the end of the culture (12 h). Indeed, pre-exposure to LPS – irrespective of C5a stimulation – resulted in reduction in C5aR mRNA levels (Fig. 4C), thus indicating that the LPS-induced C5aR downmodulation is exerted at transcriptional level. Of note, TLR activation of whole blood also resulted in negative modulation of neutrophil C5aR expression (Fig. 4B, inset). Together, these findings indicated that increased cell sensitivity to C5a following TLR activation is not due to C5aR upregulation.

### TLR activation induces cell hypersensitivity to C5a without affecting C5a-induced Ca^2+^ mobilization

**Figure 5:**
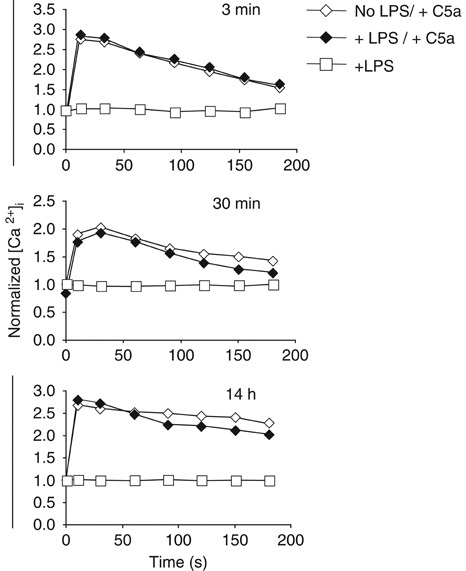
C5a-induced Ca^2+^ mobilization following PBMCs pre-exposure to LPS. C5a (10 nM)-induced changes in monocyte cell fluorescence over 180 s, as a measure of intracellular Ca^2+^ mobilization, detected by flow cytometry following PBMC (1×10^6^/condition) pre-exposure or not to LPS (100 pg/mL) for the indicated times and staining with the Ca^2+^-chelating fluorescent dye, Fluo3-AM. Background fluorescence was determined before addition of C5a (time 0) in cells pre-exposed or not to LPS. Results are expressed as normalized (Ca^2+^)_i_: the ratio between the mean fluorescence intensity at time *t* after C5a addition and that at time 0. A representative experiment out of four is shown.

Next, we tested whether the TLR modulatory effect extended to C5a-mediated intracellular Ca^2+^ mobilization, which depends on G-protein coupling to C5aR 29. PBMCs were pre-exposed to LPS for 3 min, 30 min or 14 h, or left untreated, loaded with a Ca^2+^-chelating fluorescent dye, stimulated with C5a and monitored over 3 min for changes in cell fluorescence as a measure of intracellular Ca^2+^ mobilization (Fig. 5). Following C5a stimulation, cells pre-exposed or not to LPS for any of the indicated periods of time showed similar Ca^2+^ increases (kinetics and intensity). Similar results (not shown) were obtained following cell pre-exposure to a ligand for TLR2 (Pam_3_-Cys-Ser-Lys_4_), a TLR documented to induce Ca^2+^ mobilization, unlike TLR4 30. These findings suggested that TLRs exert positive modulation on cell sensitivity to C5a by affecting a G protein-dependent event separate from Ca^2+^ mobilization or a G protein-independent signalling pathway used by C5aR.

### TLR activation reduces C5L2 receptor activity and expression

**Figure 6:**
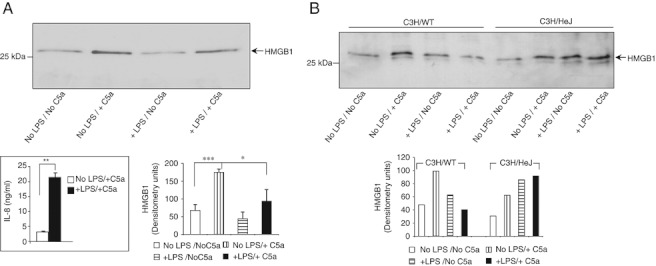
Effect of TLR activation on C5L2 receptor activity. (A) Western blot analysis and densitometric scanning of HMGB1 levels in cytoplasmic cell extracts of PBMCs (0.5×10^6^/condition) pre-exposed (14 h) or not to LPS (100 pg/mL) and subsequently activated (14 h) or not with C5a (10 nM). The IL-8 levels in the culture supernatants of this experiment are shown in the lower left inset. Results are of one experiment representative of five. ^*^p<0.05; ^**^p<0.01; ^***^p<0.005, paired Student's *t*-test. (B) Western blot analysis and densitometric scanning of C5a (60 nM)-induced levels of HMGB1 in culture supernatants of whole blood cells (100 μL whole blood/condition) from TLR4 signalling-deficient (C3H/HeJ) and WT mice (*n*=5/condition) challenged (1 h) i.p. with LPS (50 μg/mouse) or PBS (No LPS). Results shown for each condition are of the pooled supernatants of the five mice.

Seven-transmembrane receptors, like those for C5a, also signal through a G-protein-independent pathway that involves the activity of β-arrestins – multifunctional adapter proteins that mediate signalling and also control receptor desensitization and trafficking 13, 31, 32. Notably, C5aR signalling through the β-arrestin pathway was reported to be negatively modulated by the G protein-uncoupled C5a receptor, C5L2 13. Thus, to evaluate the possibility that a C5a-triggered G-protein-independent signalling event was the target of TLR modulation, we tested the effect of TLR activation on the activity of C5L2. PBMCs were pre-exposed to LPS and subsequently stimulated with C5a. Following stimulation, the cell culture supernatants were tested for IL-8 levels, and cytoplasmic cell extracts for levels of high-mobility group box-1 protein (HMGB1). HMGB1 is a nuclear factor that acts as a mediator of inflammation and sepsis whose cytoplasmic mobilization and release upon C5a stimulation depends on C5L2, but not C5aR 11, 33, 34 (Fig. 6A). Cell pre-exposure to LPS resulted in relatively lower levels of C5a-induced HMGB1 (Fig. 6A, +LPS/+C5a versus No LPS/+C5a), suggesting that TLR activation negatively affects C5L2 activity. This was in contrast to the positive modulatory effect exerted by TLRs on the C5aR-mediated pro-inflammatory responses described above and confirmed in this experiment, as the culture supernatants of PBMCs pre-exposed to LPS showed substantially higher levels of IL-8 upon C5a stimulation (Fig. 6A, inset). Further support for the role of TLRs as negative modulators of C5L2 activity was obtained from the comparative analysis of HMGB1 levels in blood cell culture supernatants between TLR4 signalling deficient and WT mice stimulated with C5a after LPS challenge in vivo. Indeed, the level of C5a-induced HMGB1 released by blood cells of WT mice exposed to LPS was lower than that of WT animals that were not exposed to LPS (Fig. 6B, C3H/WT, +LPS/+C5a versus No LPS/+C5a), whereas TLR4 signalling-deficient mice did not show these differences.

**Figure 7:**
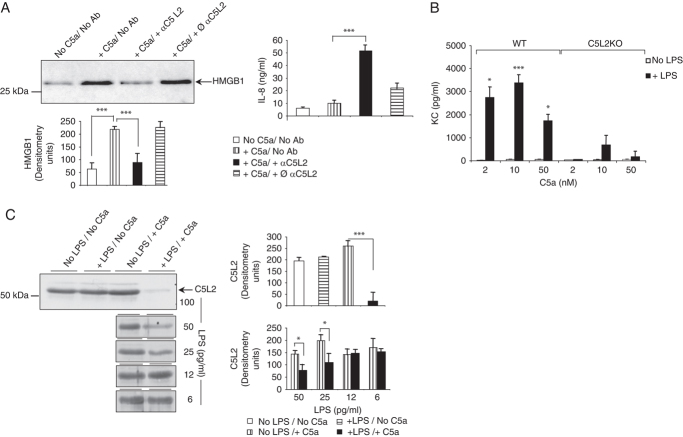
C5L2 receptor expression and cell sensitivity to C5a. (A) HMGB1 (left) and IL-8 (right) levels detected in cytoplasmic cell extracts and culture supernatants respectively, following PBMC (0.5×10^6^/condition) stimulation (14 h) with C5a (2.5 nM) in the absence or presence of an anti-C5L2 blocking mAb (5 μg/mL) or the same mAb denatured by boiling (Ø). Results are from one experiment representative of four (HMGB1) and three (IL-8). ^***^p<0.005, paired Student's *t*-test. (B) Levels of KC in blood cell culture supernatants (100 μL whole blood/condition) of C5L2KO and WT mice challenged (1 h) i.p. with LPS (50 μg/mouse) or PBS (no LPS) and stimulated (14 h) ex vivo with C5a. C5a-induced KC concentrations were estimated by background subtraction as described for Fig. 1. Values are expressed as the mean+SEM (*n*=5/condition). ^*^p<0.05, ^***^p<0.005, (LPS-treated versus no LPS, paired Student's *t*-test). (C) Western blots analysis of C5L2 levels in cell lysates of PBMCs (0.5×10^6^/condition) pre-exposed or not to LPS and subsequently activated with C5a (10 nM). Results are representative of three experiments. Densitometry of Western blots are shown in A and C; ^*^p<0.05, ^***^p<0.005, paired Student's *t*-test.

The results of C5L2 receptor blocking experiments were also consistent with the concept that the second C5a receptor is a target of TLR modulation. C5a stimulation of PBMCs in the presence of an anti-C5L2 blocking mAb showed a marked reduction in HMGB1 levels (Fig. 7A, left). By contrast, the culture supernatants of the C5L2 mAb-treated PBMCs showed higher levels of IL-8 (Fig. 7A, right). These findings confirmed previous observations on the effect of C5L2 receptor blockade on HMGB1 and cytokine production 11. Notably, the effects of C5L2 blockade were similar to those resulting from cell pre-exposure to LPS shown above (Fig. 6A), suggesting that the positive effect of TLRs on C5a-induced responses may involve inhibition of C5L2 activity. To test this possibility further, we compared blood cell sensitivity to C5a ex vivo between C5L2-deficient (C5L2KO) and WT mice that had been challenged with LPS (Fig. 7B). The C5a-induced release of KC was extremely low in both WT and C5L2KO mice not challenged with LPS. Pre-exposure to LPS resulted in a marked increase in cell sensitivity to C5a in WT but not in C5L2KO mice, indicating that C5L2 is involved in the TLR modulatory effect.

To explore the mechanism by which TLRs modulate C5L2 receptor activity, the C5L2 expression levels in cell lysates of human PBMCs pre-exposed or not to LPS and stimulated with C5a were compared (Fig. 7C). Following C5a stimulation, C5L2 levels were slightly increased. However, cell pre-exposure to LPS before C5a stimulation resulted in a marked reduction in C5L2 expression in a LPS dose-dependent manner, indicating that TLR activation negatively modulates C5L2 activity, at least in part, by reducing C5L2 expression. It is noteworthy, however, that this inhibitory effect of LPS occurs only upon subsequent C5a stimulation.

## Discussion

TLRs and the complement system play major roles in the innate immune response against microbial pathogens. Their activation triggers potent pro-inflammatory responses and microbial killing mechanisms that ensure a prompt and efficient clearance of infection. A mutually regulated and concerted activity of these two innate immune components would strengthen the efficiency of innate host defense. In support of this possibility, synergistic effects between TLRs and C5aR, and a strong regulation of TLR-mediated pro-inflammatory and immunoregulatory responses by complement receptors have been reported 17–23. These findings suggested crosstalk between TLRs and complement receptors 3, 4, 11, 20–23. However, the putative modulatory effect exerted by TLRs on complement receptor-mediated pro-inflammatory responses and the underlying mechanism have not been directly investigated. In this study, we focused on the interaction of the complement anaphylatoxin C5a with its receptors, C5aR and C5L2, and demonstrated that TLR activation exerts substantial positive modulation on C5a-induced pro-inflammatory cell responses to microbial components and whole bacteria in vitro and ex vivo. Furthermore, we presented evidence indicating that TLR modulates C5a-induced responses by negatively modulating the activity of the second C5a receptor, C5L2, which itself can act as a negative modulator of C5aR-mediated responses 11, 13, 26–29. This negative effect on C5L2 involves reduction of C5L2 receptor expression. These findings indicate the existence of genuine crosstalk between TLRs and complement that involves C5L2 and that the positive modulation of C5a-induced pro-inflammatory responses by TLR activation is a physiological feature that contributes to the prompt and efficient innate immune response against microbial pathogens.

The modulatory capacity of TLRs does not appear to be restricted by the nature of the pathogen, as we demonstrated that ligands activating a variety of TLRs are capable of inducing hypersensitivity to C5a. Notably, pre-exposure to a wide range of TLR ligand concentrations, even to concentrations well below those found in sepsis patients, resulted in enhanced responses to C5a (Fig. 1C). This finding indicates that TLR modulation of cell responses to C5a is an extremely sensitive mechanism that might operate during the course of mild as well as severe infections. However, TLR positive modulatory activity appears to be tightly controlled, as it was accompanied by a marked TLR-induced downregulation of C5aR expression and reduced C5a-induced mobilization of the late mediator of inflammation, HMGB1. This latter effect most likely resulted from the negative effect of TLR activation on C5L2 – previously demonstrated to be the receptor for C5a that mediates release of HMGB1 11. Of note, increased levels of HMGB1 were observed in the culture supernatants from blood cells of C3H/HeJ mice exposed to LPS that were not stimulated with C5a ex vivo (Fig. 6B, C3H/HeJ, +LPS/No C5a). The lack of the postulated negative modulation of C5L2 activity by TLR4 activation in these mice may have led to the observed increase in HMGB1, which most likely was generated as a consequence of LPS-induced complement activation in vivo and the resulting generation of C5a, which leads to C5L2-mediated induction and late release of HMGB1. This DNA-binding protein behaves as a potent pro-inflammatory cytokine following its late release from activated or necrotic cells 33, 34. Indeed, it has been demonstrated that HMGB1 acts as a late mediator of sepsis and endotoxin lethality, is increased in the plasma of septic patients and its blockade improves survival of septic rodents 34–38. Thus, although TLR activation imparts higher sensitivity to cellular responses to C5a, this positive modulatory effect appears to be counterbalanced by modulation of the pro-inflammatory activities of C5a and HMGB1 through the simultaneous negative effect on C5aR and C5L2 respectively. In line with the regulatory mechanism postulated here, it has been demonstrated that the combined blockade of C5aR and C5L2 greatly improved survival in a mouse model of severe sepsis 11.

This study has also shed light on the mechanism underlying the TLR modulatory effect. The TLR-mediated hypersensitivity to C5a did not result from C5a receptor upregulation, as discussed above. Furthermore, a TLR modulatory effect of similar intensity was observed over a wide range of LPS concentrations (0.1–1000 ng/mL, Fig. 1C). This, together with the observation that a 30-min cell pre-exposure to LPS was sufficient to achieve maximal hypersensitivity to C5a (Fig. 4A), suggested that modulation did not result from a carryover effect of TLR-induced cytokines. Consistent with this possibility, cells pre-exposed to LPS and subsequently cultured in the absence of C5a did not show activation of the transcription factor NF-κB – a key regulator of cytokine/chemokine gene transcription – at the end of the culture (Fig. 1F). This observation also indicated that the TLR enhancing effect did not result from residual TLR ligands carried over from the TLR activation phase.

The findings described previously, and the fact that TLR modulation did not affect Ca^2+^ mobilization, prompted us to test whether a C5a-triggered G-protein-independent signalling event was the primary target of TLR modulation. We therefore sought to test the effect of TLR activation on the activity of the G protein-uncoupled C5a receptor, C5L2. The reduced levels of C5a-induced HMGB1 observed following cell pre-exposure to LPS in vitro and ex vivo, and the LPS-mediated downregulation of C5L2 expression – although occurring indirectly upon subsequent C5a stimulation – confirmed C5L2 as a target for negative modulation by TLRs. In neutrophils, activation of C5L2 appears to occur only as a consequence of C5aR activation 13. If this was also the case in monocytes, it may be speculated that the marked downregulation of C5aR expression induced by TLR activation observed in this study contributes to the TLR negative modulatory effect on C5L2 activity. C5L2 was demonstrated to bind C5a with high affinity, similar to that of C5aR. However, in contrast to C5aR, it is unable to couple to intracellular G proteins and induce Ca^2+^ mobilization 26. Consistent with these findings, it has been demonstrated that C5L2 can act as a decoy or scavenger C5a receptor, controlling C5aR activity 26–28. It has also been reported that C5L2 negatively modulates C5aR signalling by inhibiting C5aR-β-arrestin-mediated ERK1/2 activation 13. In contrast, a number of reports have demonstrated positive or dual signalling functions of C5L2 11, 12, 39, 40. In particular, the work by Rittirsch et al. 11 demonstrated that the absence of C5L2 in mice subjected to cecal ligation and puncture (CLP) resulted in decreased levels of IL-1β, MIP-1α, MIP-2 and HMGB1, suggesting pro-inflammatory activity by C5L2. Notably, however, the same work showed that in C5L2KO mice subjected to cecal ligation and puncture, the levels of the pro-inflammatory cytokine IL-6 were significantly increased. These findings suggest that the mechanism regulating pro-inflammatory mediator release involving C5L2 is complex and that the net result of C5L2 activity may depend on the magnitude of the C5L2 modulatory effect on each mediator affected and the relative contribution of that mediator to the net resulting effect. In the present study, the similarities between the effects of C5L2 receptor blockade and those resulting from cell pre-exposure to LPS, and the lack of response of C5L2KO mice to pre-exposure to LPS (no increased sensitivity to C5a) led us to conclude that TLRs may enhance cell sensitivity to C5a by regulating the negative modulatory capacity of C5L2 on a number of pro-inflammatory mediators.

Notably, the C5a-stimulated blood cells from C5L2-deficient mice that had not been challenged with LPS did not show increased KC production. This was unexpected, as we speculated that the absence of the putative negative regulator of C5aR, C5L2, should result in increased cell sensitivity to C5a. It is possible that an additional, compensatory, negative regulatory mechanism controlling responses to C5a operates only in the complete absence of C5L2 (C5L2KO mice). This would be compatible with the increased sensitivity to C5a resulting from C5L2 receptor inhibition by TLR activation or Ab blockade observed in this study, as such a compensatory negative regulatory mechanism would not be operational due to the presence of C5L2. In contrast to C5L2, the second regulatory mechanism would not be susceptible to negative regulation by TLRs, as the blood cells from C5L2KO mice pre-treated with LPS did not show increased KC production (Fig. 7B, C5L2KO+LPS).

We observed that the degree of the TLR-induced enhancing effect on cell sensitivity to C5a varies among individuals (Fig. 2B). This may be due to the differing extent of the downmodulatory effect exerted by TLRs on C5aR and C5L2 expression, which may depend on the different constitutive expression levels of these receptors in each individual. We also observed a decline in most individuals' response to TLR activation at higher concentrations of C5a (Fig. 2B). It is possible that at higher C5a concentrations, such as those that might be generated in vivo during an acute infection, a more pronounced activation and ligand-induced downregulation of C5aR may lead to C5L2 becoming comparatively more engaged. This may result in a stronger negative modulatory effect on C5aR responses that would counteract and limit the TLR enhancing effect. Thus, an individual's capacity to clear infections efficiently and successfully resolve inflammation might be determined, at least in part, by the extent of the TLR enhancing effect relative to C5L2's capacity to counteract this positive effect.

In conclusion, the findings reported in this study demonstrate the existence of an efficient immunomodulatory network involving two major components of the innate immune system, TLRs and complement. In particular, we show that the positive modulation of TLR-mediated pro-inflammatory responses by complement receptors reported previously is paralleled by an equally substantial enhancing effect of TLRs on cell sensitivity to the pro-inflammatory peptide, C5a, through TLR negative modulation of the C5aR activity regulator, C5L2. The description of the mutually regulated and concerted activities of TLRs and complement in innate host defense may help to identify new therapeutic targets to control acute and chronic inflammatory conditions.

## Materials and methods

### Cell activations

Human blood samples were obtained from healthy volunteers as approved by the local Research Ethics Committee. PBMCs were obtained through Ficoll density-gradient centrifugation. For cell activation experiments, triplicate cell aliquots (1.5×10^5^ cells/well, unless stated otherwise) were cultured in RPMI 1640 medium (Invitrogen) supplemented with 10% heat-inactivated (56°C, 30 min) FCS (HyClone; <0.06 U/mL endotoxin) and 2 mM glutamine (complete medium), and stimulated at 37°C for 14 h or the time indicated with optimal concentrations of ultra-pure LPS (*E. coli* O111:B4 strain), zymosan, flagellin, imiquimod – all from Invivogen – Pam_3_-Cys-Ser-(Lys)_4_ HCl (Pam_3_Cys; EMC microcollections GmbH) as indicated, or medium alone (mock stimulation). Following incubation, cells were washed (3×, RPMI 1640 medium), resuspended in complete medium and activated for a further 12–14 h with the indicated concentrations of human recombinant C5a (kindly provided by Dr P.N. Monk, Sheffield University, UK) or mock activated. Cell culture supernatants were then tested for IL-8 or IL-6 by ELISA (Duoset, R&D Systems). For C5L2 receptor blocking experiments, PBMCs were preincubated (30 min at 37°C) with the anti-human C5L2 blocking mAb, 1D9-M12 (5 μg/mL; Biolegend), before stimulation with C5a (2.5 nM). In control experiments, the 1D9-M12 mAb was denatured by boiling for 10 min. For the experiments shown in Fig. 2, triplicate samples of heparinized (10 IU/mL) human whole blood (100 μL/well) were exposed for 14 h to LPS (500 pg/mL) or heat-killed *E. coli* (O111:B4 strain, 1×10^8^ CFU/mL). Subsequently, samples were centrifuged (300×*g*, 5 min), the blood cells washed (×3, RPMI 1640 medium), resuspended in heat-inactivated 100% autologous plasma and activated with the indicated concentrations of C5a.

The C5a-induced IL-8 concentrations were estimated by subtracting the background levels of IL-8 present in cultures not activated with C5a and pre-exposed or not to TLR ligands from the corresponding C5a-activated samples (background levels of the experiments described in Fig. 1B – typical of all experiments – are shown in the figure legend).

### Quantitative RT-PCR (RT-qPCR)

PBMCs (1×10^6^ cells/condition) were cultured in complete medium, stimulated or not with 100 pg/mL LPS, washed, and activated with C5a (10 nM), as described above. RNA was phenol extracted (Tri Reagent, Ambion) and reverse transcription was performed using random primers (High Capacity cDNA Reverse Transcription, Applied Biosystems). qPCR was performed on the resulting cDNA using the Power SYBR Green PCR master mix (Applied Biosystems) and specific primers (Invitrogen): IL-6, 5′-CAGTTCCTGCAGAAAAAGGC-3′ and 5′-GAATGAGATGAGTTGTCATG-3′; IL-8, 5′-GAACTGAGAGTGATTGAGAGTGGA-3′ and 5′-CTCTTCAAAAACTTCTCCACAACC-3′; C5aR, 5′-GGAGACCAGAACATGAACTC-3′ and 5′-ATCCACAGGGGTGTTGAGGT-3′; β-glucuronidase, 5′-TCTGTATTCATTGGAGGTGC-3′ and 5′-AAGGTTTCCCATTGATGAGG-3′. PCR was carried out using the ABI 7900HT real-time PCR system (Applied Biosystems), and results were analyzed by the ΔΔCt method 41.

### NF-κB assays

PBMCs (1×10^7^ cells/condition) were pre-exposed or not to 100 pg/mL LPS, washed and activated with C5a (10 nM) as previously described. Nuclear extracts were prepared (Nuclear Extract kit, Active Motif), and their protein concentration determined (ProStain Fluorescent Protein Quantification, Active Motif). Five micrograms of total protein/sample were used to determine NF-κB p65 concentrations (ELISA, TransAM NF-κB p65, Active Motif).

### In vivo model of TLR activation

Inbred 8- to 12-wk-old C3H/HeN, C3H/HeJ (Harlan), BALB/c (The Jackson Laboratory) and C5L2KO (on BALB/c background) mice were maintained under barrier conditions and pathogen free. All experimental procedures were carried out under Home Office (UK) or the Ministerium für Landwirtschaft, Umwelt und ländliche Räume (Kiel, Germany) project licenses. Mice (*n*=5/condition) were i.p. injected with a previously defined dose of LPS (50 μg/mouse) or phosphate-buffered saline (PBS). After 1 h, blood was collected by cardiac puncture and samples (100 μL/condition) were washed (×3, RPMI 1640), resuspended in complete medium and stimulated (14 h) with the indicated concentrations of mouse C5a (Hycult). The cell culture supernatants were tested for KC and MCP-1 by ELISA (R&D Systems), and for HMBG1 by Western blot.

### C5aR cell-surface expression

PBMCs (1×10^6^/condition) or whole blood (100 μL/condition) were stimulated (30 min) with 100 pg/mL (PBMCs) or 500 pg/mL (whole blood) LPS or mock stimulated. Following incubation, cell aliquots were collected for C5aR expression analysis, and the remaining samples were washed and activated or not with C5a (10 nM) for the indicated times. At each time point, cell aliquots were tested for C5aR cell-surface expression on gated monocytes or neutrophils – identified by their CD14^+^ staining and forward and side scatter profiles – by flow cytometry using a human C5aR-specific mAb (S5/1, Hycult), as described 42.

### Ca^2+^ mobilization assays

Ca^2+^ mobilization in gated monocytes was analyzed by flow cytometry, as described 43. PBMCs (1×10^6^ cells/condition) were activated or not with LPS for 30 min or 14 h before staining (30 min, room temperature) with the Ca^2+^-chelating fluorescent dye Fluo3-AM (10 μM, Molecular Probes) or first stained with Fluo3-AM before a 3-min activation with LPS. An aliquot was taken from each sample for testing the background fluorescence at time 0. C5a (10 nM) was subsequently added to the remaining samples, and fluorescence was first measured 10 s after addition of C5a, and thereafter every 30 s for a total of 3 min. Results are expressed as normalized [Ca^2+^]_i_, as a measure of the fold increase in intracellular Ca^2+^ concentration at each time point after the addition of C5a, by determining the ratio between the mean fluorescence intensity at time *t* and that at time 0.

### Western blots

HMGB1 and C5L2 levels in cytoplasmic preparations from PBMCs and in culture supernatants (20 μL) of mouse blood cells (HMGB1) were evaluated by Western blot analysis, as described 44. Here, PBMCs (0.5×10^6^ cells/condition) were cultured with or without LPS, washed and stimulated or not with C5a. Cells were then lysed (0.5% v/v Nonidet P-40, 50 mM Tris-HCl, 150 mM NaCl, 1 μg/mL leupeptin and pepstatin, 1 mM PMSF, pH 7.4 buffer) for 1 h on ice, and the protein content of the cytoplasmic cell extracts was estimated (BCA assay, Bio-Rad). HMGB1 and C5L2 were detected by using an anti-human and mouse HMGB1-specific polyclonal Ab (Ab18256, Abcam) and the anti-human C5L2 mAb 1D9-M12.

### Statistical analysis

Statistical analysis of the data was performed by using a paired Student's *t*-test. *p* values <0.05 were considered significant.

## Abbreviation

KC: Keratinocyte-derived chemokine
HMGB1: highmobility group box-1 protein

## References

[1] Kumar H, Kawai T, Akira S (2009). Toll-like receptors and innate immunity. Biochem. Biophys. Res. Commun.

[2] Walport MJ (2001). Complement. First of two parts. N. Engl. J. Med.

[3] Hajishengallis G, Lambris JD (2010). Crosstalk pathways between Toll-like receptors and the complement system. Trends Immunol.

[4] Ricklin D, Hajishengallis G, Kun Yang K, Lambris JD (2010). Complement: a key system for immune surveillance and homeostasis. Nat. Immunol.

[5] Gay NJ, Gangloff M (2007). Structure and function of Toll receptors and their ligands. Annu. Rev. Biochem.

[6] Liew FY, Xu D, Brint EK, O'Neill LA (2005). Negative regulation of toll-like receptor-mediated immune responses. Nat. Rev. Immunol.

[7] Kanzler H, Barrat FJ, Hessel EM, Coffman RL (2007). Therapeutic targeting of innate immunity with Toll-like receptor agonists and antagonists. Nat. Med.

[8] Mollnes TE, Song WC, Lambris JD (2002). Complement in inflammatory tissue damage and disease. Trends Immunol.

[9] Guo RF, Riedemann NC, Ward PA (2004). Role of C5a-C5aR interaction in sepsis. Shock.

[10] Ward PA (2004). The dark side of C5a in sepsis. Nat. Rev. Immunol.

[11] Rittirsch D, Flierl MA, Nadeau BA, Day DE, Huber-Lang M, Mackay CR, Zetoune FS (2008). Functional roles for C5a receptors in sepsis. Nat. Med.

[12] Chen NJ, Mirtsos C, Suh D, Lu YC, Lin WJ, McKerlie C, Lee T (2007). C5L2 is critical for the biological activities of the anaphylatoxins C5a and C3a. Nature.

[13] Bamberg CE, Mackay CR, Lee H, Zahra D, Jackson J, Lim YS, Whitfeld PL (2010). The C5a receptor C5L2 is a negative modulator of C5aR-mediated signal transduction. J. Biol. Chem.

[14] Mollnes TE, Christiansen D, Brekke OL, Espevik T (2008). Hypothesis: combined inhibition of complement and CD14 as treatment regimen to attenuate the inflammatory response. Adv. Exp. Med. Biol.

[15] Riedemann NC, Guo RF, Ward PA (2003). Novel strategies for the treatment of sepsis. Nat. Med.

[16] Raby AC, Le Bouder E, Colmont C, Davies J, Richards P, Coles B, George CH (2009). Soluble TLR2 reduces inflammation without compromising bacterial clearance by disrupting TLR2 triggering. J. Immunol.

[17] Riedemann NC, Guo RF, Sarma VJ, Laudes IJ, Huber-Lang M, Warner RL, Albrecht EA (2002). Expression and function of the C5a receptor in rat alveolar epithelial cells. J. Immunol.

[18] Laudes IJ, Chu JC, Huber-Lang M, Guo RF, Riedemann NC, Sarma JV, Mahdi F (2002). Expression and function of the C5a receptor in mouse microvascular endothelial cells. J. Immunol.

[19] Riedemann NC, Guo RF, Hollmann TJ, Gao H, Neff TA, Reuben JS, Speyer CL (2004). Regulatory role of C5a in LPS-induced IL-6 production by neutrophils during sepsis. FASEB J.

[20] Zhang X, Kimura Y, Fang C, Zhou L, Sfyroera G, Lambris JD, Wetsel RA (2007). Regulation of Toll-like receptor-mediated inflammatory response by complement *in vivo*. Blood.

[21] Weaver DJ, Reis ES, Pandey MK, Köhl G, Harris N, Gerard C, Köhl J (2010). C5a receptor-deficient dendritic cells promote induction of Treg and Th17 cells. Eur. J. Immunol.

[22] Hawlisch H, Belkaid Y, Baelder R, Hildeman D, Gerard C, Köhl J (2005). C5a negatively regulates toll-like receptor 4-induced immune responses. Immunity.

[23] Wang M, Krauss JL, Domon H, Hosur KB, Liang S, Magotti P, Triantafilou M (2010). Microbial hijacking of complement-toll-like receptor crosstalk. Sci. Signal.

[24] Opal SM, Scannon PJ, Vincent JL, White M, Carroll SF, Palardy JE, Parejo NA (1999). Relationship between plasma levels of lipopolysaccharide (LPS) and LPS-binding protein in patients with severe sepsis and septic shock. J. Infect. Dis.

[25] Sabroe I, Jones EC, Usher LR, Whyte MK, Dower SK (2002). Toll-like receptor (TLR)2 and TLR4 in human peripheral blood granulocytes: a critical role for monocytes in leukocyte lipopolysaccharide responses. J. Immunol.

[26] Okinaga S, Slattery D, Humbles A, Zsengeller Z, Morteau O, Kinrade MB, Brodbeck RM (2003). C5L2, a nonsignaling C5A binding protein. Biochemistry.

[27] Gao H, Neff TA, Guo RF, Speyer CL, Sarma JV, Tomlins S, Man Y (2005). Evidence for a functional role of the second C5a receptor C5L2. FASEB J.

[28] Scola AM, Johswich KO, Morgan BP, Klos A, Monk PN (2009). The human complement fragment receptor, C5L2, is a recycling decoy receptor. Mol. Immunol.

[29] Johswich K, Klos A (2007). C5L2- an anti-inflammatory molecule or a receptor for acylation stimulating protein (C3a-desArg)?. Adv. Exp. Med. Biol.

[30] Chun J, Prince A (2006). Activation of Ca^2+^-dependent signaling by TLR2. J. Immunol.

[31] Lefkowitz RJ (2004). Historical review: a brief history and personal retrospective of seven-transmembrane receptors. Trends Pharmacol. Sci.

[32] Rajagopal S, Kim J, Ahn S, Craig S, Lam CM, Gerard NP, Gerard C (2010). Beta-arrestin- but not G protein-mediated signaling by the “decoy” receptor CXCR7. Proc. Natl. Acad. Sci. USA.

[33] Gardella S, Andrei C, Ferrera D, Lotti LV, Torrisi MR, Bianchi ME, Rubartelli A (2002). The nuclear protein HMGB1 is secreted by monocytes via a non-classical, vesicle-mediated secretory pathway. EMBO Rep.

[34] Wang H, Zhu S, Zhou R, Li W, Sama AE (2008). Therapeutic potential of HMGB1-targeting agents in sepsis. Expert Rev. Mol. Med.

[35] Lotze MT, Tracey KJ (2005). High-mobility group box 1 protein (HMGB1): nuclear weapon in the immune arsenal. Nat. Rev. Immunol.

[36] Suda K, Kitagawa Y, Ozawa S, Saikawa Y, Ueda M, Ebina M, Yamada S (2006). Anti-high-mobility group box chromosomal protein 1 antibodies improve survival of rats with sepsis. World J. Surg.

[37] Yang H, Ochani M, Li J, Qiang X, Tanovic M, Harris HE, Susarla SM (2004). Reversing established sepsis with antagonists of endogenous high-mobility group box 1. Proc. Natl. Acad. Sci. USA.

[38] Qin S, Wang H, Yuan R, Li H, Ochani M, Ochani K, Rosas-Ballina M (2006). Role of HMGB1 in apoptosis-mediated sepsis lethality. J. Exp. Med.

[39] Kalant D, MacLaren R, Cui W, Samanta R, Monk PM, Laporte SA, Cianflone K (2005). C5L2 is a functional receptor for acylation-stimulating protein. J. Biol. Chem.

[40] Zhang X, Schmudde I, Laumonnier Y, Pandey MK, Clark JR, König P, Gerard NP (2010). A critical role for C5L2 in the pathogenesis of experimental allergic asthma. J. Immunol.

[41] Livak KJ, Schmittgen TD (2001). Analysis of relative gene expression data using real-time quantitative PCR and the 2(-Delta Delta C(T)) Method. Methods.

[42] Rey Nores JE, Bensussan A, Vita N, Stelter F, Arias MA, Jones M, Lefort S (1999). Soluble CD14 acts as a negative regulator of human T cell activation and function. Eur. J. Immunol.

[43] Schepers E, Glorieux G, Dhondt A, Leybaert L, Vanholder R (2009). Flow cytometric calcium flux assay: evaluation of cytoplasmic calcium kinetics in whole blood leukocytes. J. Immunol. Methods.

[44] LeBouder E, Rey-Nores JE, Rushmere NK, Grigorov M, Lawn SD, Affolter M, Griffin GE (2003). Soluble forms of Toll-like receptor (TLR)2 capable of modulating TLR2 signaling are present in human plasma and breast milk. J. Immunol.

